# Examining for race-associated differences on Sway Medical System balance and cognitive tests used for sport-related concussion

**DOI:** 10.3389/fneur.2025.1547004

**Published:** 2025-08-21

**Authors:** Katie L. Stephenson, Julia E. Maietta, Nathan E. Cook, Lamont E. Cavanagh, Heidi A. VanRavenhorst-Bell, Marc A. Norman, Alicia M. Kissinger-Knox, Grant L. Iverson

**Affiliations:** ^1^College of Osteopathic Medicine, University of New England, Biddeford, ME, United States; ^2^Department of Physical Medicine and Rehabilitation, Harvard Medical School, Boston, MA, United States; ^3^Mass General for Children Sports Concussion Program, Waltham, MA, United States; ^4^Department of Physical Medicine and Rehabilitation, Spaulding Rehabilitation Hospital, Charlestown, MA, United States; ^5^Department of Family and Community Medicine, University of Oklahoma School of Community Medicine, Tulsa, OK, United States; ^6^Department of Human Performance Studies, Wichita State University, Wichita, KS, United States; ^7^Human Performance Laboratory, Wichita State University, Wichita, KS, United States; ^8^Department of Psychiatry, University of California, San Diego, San Diego, CA, United States; ^9^Department of Physical Medicine and Rehabilitation, Schoen Adams Research Institute at Spaulding Rehabilitation, Charlestown, MA, United States

**Keywords:** concussion assessment, head injuries, racial differences, traumatic brain injury, neurocognitive assessment, balance, thoracic sway

## Abstract

**Background:**

Race associated differences and disparities in test scores, such as on neuropsychological measures, can complicate the interpretation of these test scores in student athletes following a concussion. It is unknown if there are race associated differences on the Sway Medical System, a battery that includes balance and cognitive tests for use in concussion management.

**Purpose:**

To determine if there are race-associated differences in Sway Medical System balance and cognitive module scores among athletes undergoing preseason baseline testing.

**Method:**

Athletes between 12 and 22 years old were administered the Sway Medical System balance and cognitive test modules during preseason baseline testing. Individuals with a past medical history of ADHD or concussion within the past 6 months were excluded from the study. Athletes (*N* = 27,776) who self-identified as “Black or African American” or “White” were compared on Balance, Reaction Time, Inspection Time, Impulse Control, and Memory Module scores using Mann–Whitney *U* tests, and statistical tests were stratified by age and sex.

**Results:**

The race-associated differences (effect sizes) between Black and White athletes ranged from extremely small (negligible) to small across all ages for both sexes on Balance, Reaction Time, Inspection Time, and Impulse Control scores. For the Memory Module, the effect sizes ranged from small to medium across all ages for both sexes. White boys/men had higher Memory scores than Black boys/men (Hedges’ *g* = −0.18 to −0.60). White girls/women had higher Memory scores than Black girls/women (*g* = −0.13 to −0.39).

**Conclusion:**

The race-associated differences between Black and White student-athletes on Sway Medical System balance and cognitive module scores are generally negligible. The reasons for modest race-associated differences on Memory scores are unknown and future research to examine the possible role or influence of social risk factors and psychosocial factors on module scores is recommended.

## 1 Introduction

The American Academy of Pediatrics has recommended prioritizing research that facilitates the elimination of health disparities and healthcare disparities related to race, ethnicity, and socioeconomic status (1). This recommendation is relevant for sports neuropsychology and for research relating to the assessment, treatment, and rehabilitation of sport-related concussions (2–5). Understanding and addressing if disparities exist in concussion assessment tools is one step in ensuring equitable healthcare delivery in sports medicine, particularly given the documented health disparities that exist across racial and ethnic groups in various medical contexts.

Neuropsychological tests measuring attention, reaction time, processing speed, and memory are commonly used for the medical management of sport-related concussions (6–8). Researchers have reported that people who identify as Black or African American, on average, have lower scores on some neuropsychological tests—and race-associated disparities in education and socioeconomic status (SES) are among many potential factors that likely underlie differences in neuropsychological test scores between groups (9–16). In athletic populations specifically, some race-associated differences have been observed on sport-concussion assessment tools. The presence of race and SES associated differences and disparities in neuropsychological test scores can complicate the interpretation of these test scores in student athletes following a concussion, and certain social determinants of health might be important to consider during concussion assessment and medical management, including access to quality healthcare, neighborhood characteristics, and cultural factors (4).

ImPACT is a neurocognitive assessment battery designed and used in sport-concussion management, and researchers have reported race-associated differences on ImPACT such that Black student-athletes, on average, had lower verbal memory, visual memory, visual motor speed, and reaction time scores during baseline preseason testing than White student-athletes (15, 17, 18). One study examined the rates of low ImPACT test scores in high school students from a lower SES region in Ohio and found that Black student-athletes, compared to White student-athletes, were more likely to have low scores across multiple neurocognitive domains including composite scores for verbal memory, visual memory, visual motor speed, and reaction time. This study found that the observed differences appeared to be primarily associated with SES rather than race per se (15). Moreover, in that study both the White and the Black student-athletes had more low scores than a national sample of adolescents who took ImPACT (19), which emphasizes the importance of considering SES variables when examining observed racial differences in test scores on ImPACT (15).

Sway Medical System Balance and Cognitive Modules (20) is another assessment battery designed and used in the medical management of sport-related concussion. Sway modules are administered via a smartphone or iPad application and are designed to measure balance and cognitive functioning (21–23). Balance assessment is an important component of concussion evaluation because balance impairments are relatively common following a concussion (24, 25). The Sway Balance module utilizes the smartphone’s built-in triaxial accelerometer to measure postural sway. The stances used are very similar, but not identical to, the stances included in the Balance Error Scoring System (26, 27). The Sway application also includes several cognitive tests. There are two methods for interpreting Sway test results. First, normative reference values are provided for the Sway scores. Second, Sway can be used for baseline preseason testing and student-athletes’ baseline scores can be compared to their post-injury scores. Of course, a clinician can use both methods to interpret post-injury test results when preseason scores are available. There are age and sex-associated differences in Sway balance and cognitive module scores, so the normative reference values built into the Sway application are stratified by age and sex. However, whether there are differences in Sway balance and cognitive module scores associated with race is unknown.

The purpose of this study was to determine if there are race-associated differences in Sway Medical System Balance and Cognitive Module scores in student athletes undergoing preseason baseline testing. This is important because if race-associated differences are present, then clinicians would need to determine whether those differences are large enough that they need to be considered, somehow, when interpreting post-injury Sway test scores during the medical management of concussion. If race-associated differences exist in baseline scores, this could create interpretive challenges with accurately assessing trajectories of decline and recovery following injury when pre-injury baseline performance differs systematically between groups. Moreover, if pronounced race-associated differences are present, then additional research would be needed to deconstruct those differences and try to identify social determinants of health and social psychological factors that might contribute to those differences. Given that race-associated differences have been reported on ImPACT (15, 17, 18, 28) and other neuropsychological tests (29–31), we hypothesized that student-athletes who self-identified as Black or African American would have lower scores, on average, than those who identified as White on the Sway cognitive modules. We hypothesized that there would be no race-associated differences in the Sway Balance module scores. A secondary aim was to compare the magnitude of race-associated effect sizes with sex-associated effect sizes to provide context for the clinical significance of any observed race-associated differences and to facilitate comparison with the well-established sex differences that are already incorporated into Sway’s normative framework.

## 2 Materials and methods

### 2.1 Participants and procedures

Deidentified data from 44,045 student-athletes between 12 and 22 years old who completed preseason baseline testing between July and October 2022 were provided by the company that distributes Sway. The Sway Medical System (version Sport+) was administered as part of routine preseason baseline testing protocols at schools and sports organizations. Testing was typically conducted in school gymnasiums, training facilities, or similar environments by trained personnel (coaches, athletic trainers, or healthcare providers) who had received standardized instructions on Sway administration. The complete battery takes approximately 15–20 min to administer and consists of the same standardized protocol for all ages (12–22 years). Each person completed the testing battery once and received standardized instructions through the app interface before beginning each component assessment. The Sway Medical System does not have performance validity indicators.

Students self-report demographic history using the Sway app prior to completing baseline testing. We used this self-reported demographic data to make the following exclusions to the sample. Students who self-reported having been diagnosed with ADHD (*n* = 5,610), who had missing data for ADHD status (*n* = 3,376), or who reported having sustained a concussion within the past 6 months (*n* = 378) were excluded. After making these exclusions, there were 34,154 eligible participants in the database.

In the Sway app, the student-athletes were given the option to choose a single race among the following options: White, Black or African American, Asian, American Indian or Alaska Native, Native Hawaiian or Other Pacific Islander, or “other,” which was undefined. African American is a term that refers to American people who are of African ancestry and it relates to ethnicity though is also commonly used as a term for race. African American and Black are not synonymous. We have assumed that youth choosing this race category self-identified as African American, Black, or both. In this paper, we refer to this racial category as “Black.” Of the 34,154 eligible participants in the database, 23,065 participants self-selected their race as “White,” 4,711 participants self-selected their race as “Black or African American,” 1,557 as “Asian,” 438 as “American Indian or Alaska Native,” 359 as “Native Hawaiian or Other Pacific Islander,” and 3,934 self-selected their race as “Other.” Due to small sample sizes, we chose to focus this study on comparing race-associated differences in the two largest groups, those who self-identified as Black or African American and those who identified as White. These are also the two groups that have been most often compared in prior studies. We recognize the need for future research to explore race associated differences in a diverse group of races, and we have plans to pursue that work. The final sample consisted of 27,776 individuals who identified as White or Black who took the Sway Medical System as part of their regular preseason sports participation assessment.

Additionally, in the Sway app, student-athletes are given the option to choose their “sex” as either “male” or “female.” They are also provided a question relating to their gender, and they were given the following options: “male,” “female,” “non-binary,” and “prefer not to disclose.” We analyzed the “sex” variable because that is the variable used for the Sway normative reference values. We report the gender identity of the sample separately as part of the sample description.

### 2.2 Measures

The Sway Medical System is an assessment comprised of four modules including a demographics/medical history section, symptom questionnaire, postural sway testing, and cognitive testing. Postural sway is measured by having the participant assume five stances from the Balance Error Scoring System test (i.e., feet together, tandem stance with left in front, tandem stance with right in front, left single leg stance, and right single leg stance) for 10 s in each stance. The composite Balance score, with scores ranging from 0 to 100, represents how consistently the participant remained in the steady starting position during the test. The higher the scores, the better their balance and more stable their stances.

The four cognitive modules are Reaction Time, Impulse Control, Inspection Time, and Memory. For Reaction Time, a measure of simple visual motor reaction time, participants hold the phone or tablet in their hands and are instructed to tip the device forward as soon as the screen changes from white to orange. Reaction Time is measured in milliseconds, with lower scores representing better performance. The Impulse Control Module, requiring choice reaction time within a go-no/go test, is a go/no-go task in which athletes either move or do not move the mobile device. Impulse Control is measured as the average length of time in milliseconds it takes to move the device for “go” stimuli only, and a lower score is considered better. During the Impulse Control task, participants hold their device and are instructed to watch for either a green circle with a checkmark or a red circle with an “X” in the middle. If they are presented with the green circle, they are to move the device in any direction. If they are presented with the red circle, they are to keep the device still. The Inspection Time Module, requiring simple visual inspection speed, presents athletes with two T-shaped lines for a short period of time before the lines are obscured. The participant is required to select which line was longer. The duration of time that the lines are presented is gradually reduced as the participant correctly identifies the longer line. Similar to Reaction Time, Inspection Time is measured in milliseconds, with lower scores representing better performance. The Memory Module, requiring visual working memory, presents the athlete with three letters followed by a task in which the participant is instructed to replicate a sequence of squares that turn orange on the screen. After replicating the sequences, the participant attempts to recall the originally presented three letters. The Memory Module is scored on a zero to 100 scale with 100 being the highest possible score.

### 2.3 Statistical analyses

Standard scores (*Z* scores) for Balance and cognitive module scores (i.e., Reaction Time, Impulse Control, Inspection Time, and Memory) were calculated using means and standard deviations from official Sway normative data (32); these norms are adjusted for sex and age. Participants’ scores that were three or more standard deviations below the published normative means were considered outliers and were excluded on a pairwise basis. Due to this, there were different sample sizes for the various module scores. Sample demographics are described using frequencies, means, medians, and standard deviations where appropriate.

Race-associated differences in cognitive module scores were assessed using Mann–Whitney *U* tests to compare individuals who self-identified as White and Black within the total sample and then for exploratory purposes they were conducted separately for boys/young men and girls/young women. Hedges’ *g* effect sizes were interpreted as small = 0.2, medium = 0.5, and large = 0.8 (33). Effect sizes were emphasized in the interpretation of results given that the extremely large sample sizes will result in small differences being identified as statistically significant.

As exploratory analyses, we examined correlations between age and Balance scores via three Spearman rank order correlations, one for the total sample, and two among boys/young men and girls/young women separately. Additionally, we examined for sex differences in Balance scores using Mann–Whitney *U* tests for the total sample and for each age group (12 years old, 13 years old, 14 years old, and so on, through 22 years old). Associations between age and the four cognitive module scores (i.e., Reaction Time, Impulse Control, Inspection Time, and Memory) were estimated via 12 Spearman rank order correlations, for the total sample and then separately among boys/young men and girls/young women, respectively (three correlations for each of the four modules, totaling 12 correlation coefficients). The magnitude of Spearman rank order values was interpreted as negligible (*ρ* = 0.00–0.09), weak (*ρ* = 0.1–0.3), moderate (*ρ* = 0.4–0.6), and strong (*ρ* = 0.7–0.9) (34). We examined for sex differences on the four cognitive module scores using Mann–Whitney *U* tests, where boys/young men and girls/young women were compared within the total sample and then, for exploratory purposes, within each of the 11 age groupings. Statistical significance was set at *p* < 0.05.

## 3 Results

The final sample included 27,776 participants with an average age of 16.83 years old (*SD* = 2.45; range = 12–22). Approximately 51% of the sample self-identified as female, 48% identified as male, 0.4% identified as non-binary, and 0.3% preferred not to disclose their gender. Eighty-three percent of the sample self-identified as White (*n* = 23,065) and 17% self-identified as Black (*n* = 4,711). Information about participants’ ethnicity was not available.

### 3.1 Balance scores

**Figure 1:**
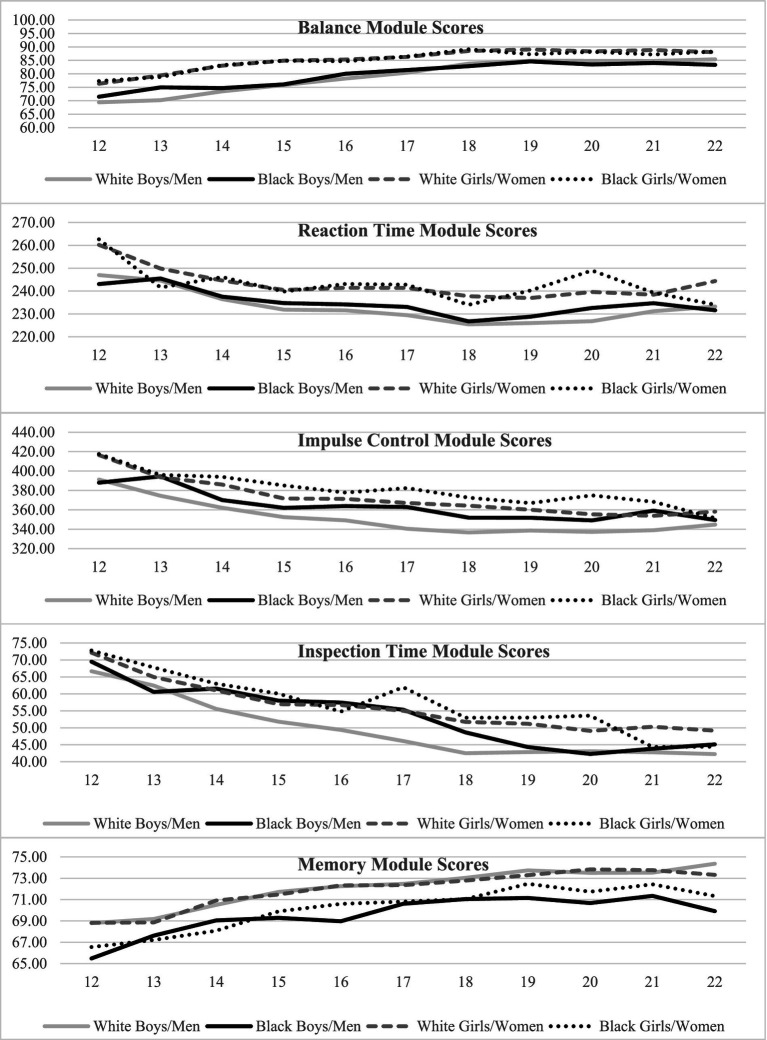
Sway balance and cognitive module scores by age, sex, and race.

**Figure 2:**
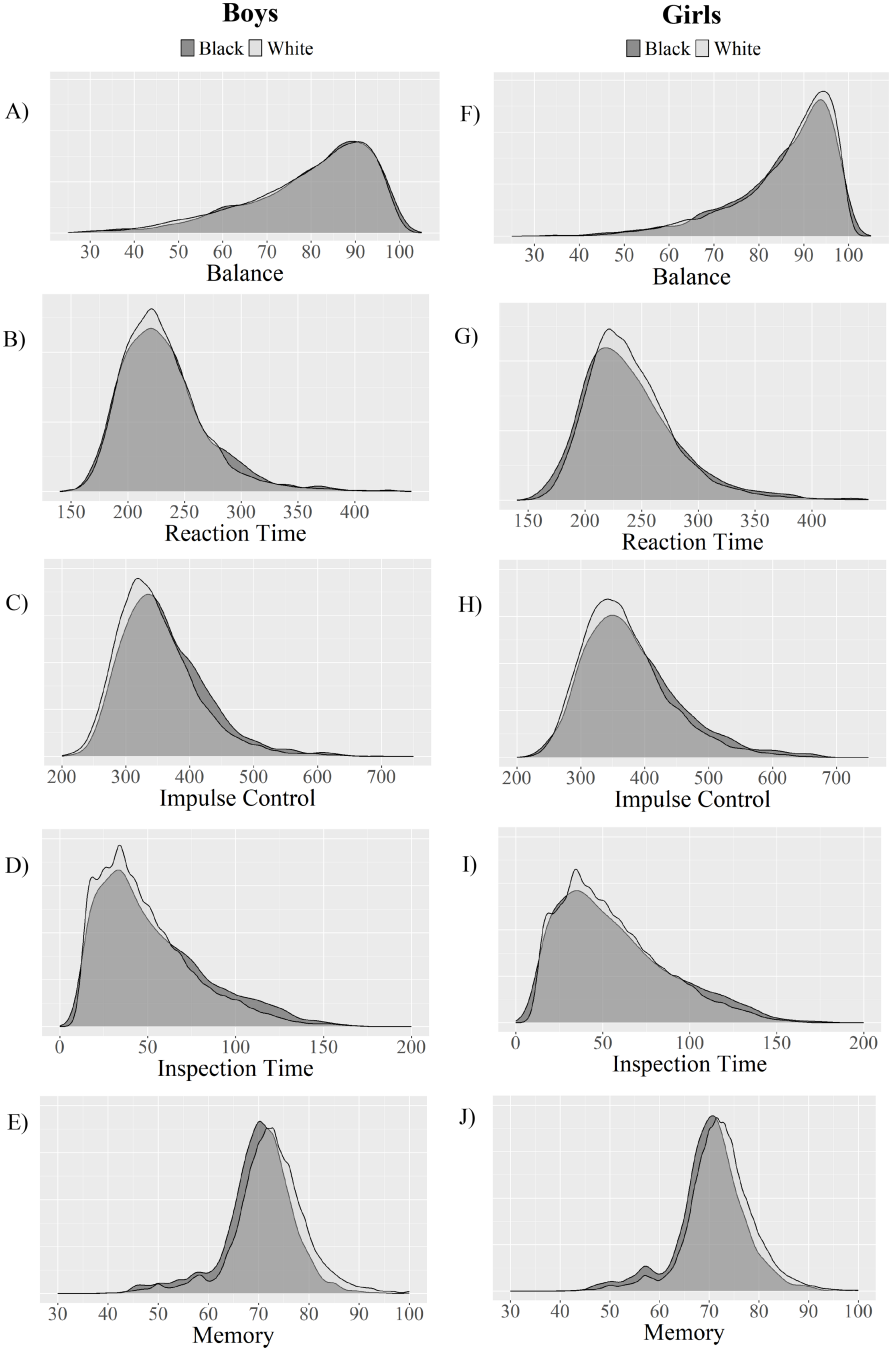
Density plots of Sway balance and cognitive module scores by sex and race. Panels **(A–E)** present overlapping density plots for Balance, Reaction Time, Impulse Control, Inspection Time, and Memory scores (respectively) for boys/men by race. Panels **(F–J)** present these data for girls/women.

Correlations between age and Balance scores are reported in Table 1. Girls and young women had higher balance scores than boys and young men in the total sample (*p < 0*.001; *g =* 0.52) and in each age group. Effect sizes for these differences were mostly small to medium (see Table 2 for balance scores by sex and age). Descriptive statistics and effect sizes comparing participants identifying as White or Black are presented in Table 3. The effect sizes ranged from extremely small (negligible) to small across all ages for both sexes (Table 3). The distributions of Balance Module scores, by race, for both sexes, are almost entirely overlapping for almost every age group. There was a small effect size difference (*g* = 0.29) among 13-year-old boys, such that White boys had higher balance scores than Black boys. There was a small effect size difference (*g* = 0.22) among 19-year-old girls, such that White girls had higher balance scores than Black girls. Figure 1 visually displays Balance scores by race and sex in each age group. Figure 2 presents overlapping density plots for Balance scores by race and by sex (boys/men in Panel A and girls/women in Panel F).

**Table 1:** Spearman correlations between age and Sway Medical Module scores within the total sample, and within boys/men and girls/women separately.

	Total sample			Boys/men			Girls/women		
	*N*	*ρ*	*p*	*N*	*ρ*	*p*	*N*	*ρ*	*p*
Balance	27,357	0.27	<0.001	13,134	0.32	<0.001	14,223	0.22	<0.001
Reaction Time	27,582	−0.06	<0.001	13,206	−0.09	<0.001	14,376	−0.05	<0.001
Impulse Control	27,475	−0.15	<0.001	13,140	−0.16	<0.001	14,335	−0.16	<0.001
Inspection Time	27,423	−0.17	<0.001	13,121	−0.21	<0.001	14,302	−0.15	<0.001
Memory	27,321	0.15	<0.001	13,089	0.14	<0.001	14,232	0.16	<0.001

**Table 2:** Sway Balance module scores by age and sex.

Ages	Boys/men				Girls/women				*p*	*g*
	*n*	*M*	Median	SD	*n*	*M*	Median	SD		
12	298	69.68	72.80	16.72	323	76.62	80.38	15.45	<0.001	**0.43**
13	557	70.94	73.93	16.24	539	79.41	82.87	13.97	<0.001	**0.56**
14	1,792	73.73	76.47	15.43	1,968	83.07	86.70	12.33	<0.001	**0.67**
15	2,343	75.90	79.39	15.08	2,168	84.97	88.02	11.18	<0.001	**0.68**
16	1,565	78.67	81.72	13.94	1,615	85.23	88.76	11.26	<0.001	**0.52**
17	1,263	80.61	83.70	12.83	1,330	86.33	88.83	9.90	<0.001	**0.50**
18	1,792	83.49	86.47	11.75	2,311	88.55	91.17	9.08	<0.001	**0.49**
19	1,601	84.80	87.57	10.87	1,773	88.89	91.35	8.39	<0.001	**0.42**
20	842	84.54	87.53	11.23	1,061	88.33	90.82	8.89	<0.001	**0.38**
21	648	84.59	87.58	11.17	760	88.67	91.54	9.19	<0.001	**0.40**
22	433	84.79	87.30	10.76	375	87.96	91.28	10.24	<0.001	**0.30**
12–22	13,134	79.43	82.87	14.23	14,223	86.06	86.48	10.95	<0.001	**0.52**

*g*, Hedges’ effect size; Effect sizes of 0.20 or greater are bolded; M, mean; SD, standard deviation; *p*, alpha from Mann–Whitney U tests.

**Table 3:** Sway Balance module scores stratified by age, sex, and self-identified race.

	Boys/men							Girls/women								
	White			Black					White			Black				
	*n*	M	SD	*n*	M	SD	*p*	*g*	*n*	M	SD	*n*	M	SD	*p*	*g*
12	237	69.21	16.71	61	71.49	16.76	0.357	0.14	277	76.50	15.56	46	77.30	14.91	0.813	0.05
13	470	70.20	16.22	87	74.96	15.81	**0.008**	**0.29**	470	79.50	13.67	69	78.81	15.98	0.870	−0.05
14	1,437	73.49	15.51	355	74.69	15.07	0.220	0.08	1,712	83.05	12.22	256	83.23	13.03	0.395	0.01
15	1,866	75.86	14.82	477	76.08	16.07	0.324	0.01	1,883	84.98	11.16	285	84.90	11.37	0.969	−0.01
16	1,190	78.25	14.14	375	80.01	13.23	0.058	0.13	1,382	85.32	11.18	233	84.67	11.74	0.453	−0.06
17	989	80.39	12.92	274	81.42	12.49	0.236	0.08	1,141	86.35	9.85	189	86.25	10.28	0.955	−0.01
18	1,360	83.72	11.64	432	82.77	12.07	0.134	−0.08	2,062	88.48	9.17	249	89.12	8.34	0.593	0.07
19	1,260	84.86	10.82	341	84.60	11.06	0.811	−0.02	1,605	89.06	8.32	168	87.25	8.91	**0.004**	**−0.22**
20	658	84.83	11.26	184	83.48	11.06	0.058	−0.12	953	88.35	8.92	108	88.18	8.58	0.627	−0.02
21	475	84.79	11.05	173	84.05	11.52	0.433	−0.07	685	88.83	9.08	75	87.19	10.06	0.208	−0.18
22	305	85.40	10.53	128	83.35	11.21	0.056	−0.19	321	88.02	10.14	54	87.64	10.91	0.857	−0.04
12–22	10,247	79.25	14.32	2,887	80.07	13.89	0.011	0.06	12,491	86.13	10.88	1,732	85.55	11.38	0.062	−0.05

*g*, Hedges’ effect size; Effect sizes of 0.20 or greater are bolded; M, mean; SD, standard deviation; *p*, alpha from Mann–Whitney U tests.

### 3.2 Cognitive scores

Correlations between age and cognitive scores for the total sample and for boys/men and girls/women are reported in Table 1. The correlations with age are small. Girls/young women were compared to boys/young men on cognitive scores in Table 4. The effect sizes between groups ranged from extremely small (negligible) to small-medium (e.g., 0.40).

**Table 4:** Sway cognitive module scores stratified by age and sex.

Ages	Boys/men				Girls/women				*p*	*g*
	*n*	*M*	Median	SD	*n*	*M*	Median	SD		
Reaction time										
12	298	246.15	242.17	46.03	320	260.55	252.00	50.63	**<0.001**	**0.30**
13	553	244.74	234.33	60.26	539	248.79	241.50	43.75	**0.002**	0.08
14	1,792	236.68	229.50	42.73	1,976	244.68	236.50	44.91	**<0.001**	0.18
15	2,356	232.33	225.42	39.47	2,201	240.62	233.50	41.12	**<0.001**	**0.21**
16	1,575	232.00	226.00	39.72	1,633	241.66	234.50	44.56	**<0.001**	**0.23**
17	1,274	230.24	224.00	40.33	1,348	241.58	235.00	39.34	**<0.001**	**0.28**
18	1,807	225.58	221.00	32.93	2,334	237.28	233.00	35.01	**<0.001**	**0.34**
19	1,612	226.60	221.50	34.27	1,801	237.26	231.67	36.95	**<0.001**	**0.30**
20	850	228.09	223.50	35.00	1,077	240.58	234.50	38.29	**<0.001**	**0.34**
21	652	232.12	227.50	37.92	767	238.51	232.00	36.82	**<0.001**	0.17
22	437	232.36	226.00	39.09	380	242.90	237.00	37.76	**<0.001**	**0.27**
12–22	13,206	231.61	225.00	39.83	14,376	241.12	234.50	40.51	**<0.001**	**0.24**
Impulse control										
12	299	390.50	376.50	74.23	320	416.61	405.25	84.19	**<0.001**	**0.33**
13	551	377.57	368.50	77.80	538	393.87	380.25	78.44	**<0.001**	**0.21**
14	1,786	363.74	352.67	67.74	1,971	387.06	374.00	73.06	**<0.001**	**0.33**
15	2,341	354.26	346.00	61.52	2,188	373.56	364.00	68.27	**<0.001**	**0.30**
16	1,574	352.65	341.50	65.79	1,627	372.05	362.00	69.78	**<0.001**	**0.29**
17	1,268	345.33	333.50	64.73	1,347	369.16	359.00	65.81	**<0.001**	**0.36**
18	1,792	340.28	331.50	56.72	2,334	365.15	356.50	66.12	**<0.001**	**0.40**
19	1,605	341.23	329.50	59.40	1,791	360.76	350.50	61.88	**<0.001**	**0.32**
20	840	339.75	330.25	56.17	1,071	357.35	347.50	63.10	**<0.001**	**0.29**
21	653	344.35	335.50	61.86	768	355.29	343.50	63.04	**<0.001**	0.17
22	431	346.16	335.50	60.14	380	357.24	344.50	65.09	**0.015**	0.18
12–22	13,140	351.11	340.83	64.09	14,335	370.96	360.33	68.97	**<0.001**	**0.30**
Inspection time										
12	300	67.17	59.50	32.93	318	72.09	68.00	33.17	**0.046**	0.15
13	553	62.16	56.67	32.99	535	65.10	59.50	33.03	0.090	0.09
14	1,788	56.77	51.00	31.59	1,967	61.30	56.67	31.99	**<0.001**	0.14
15	2,336	53.09	45.33	28.83	2,182	57.37	51.00	30.39	**<0.001**	0.14
16	1,566	51.31	42.50	29.09	1,625	56.43	51.00	29.60	**<0.001**	0.17
17	1,264	48.14	42.50	27.24	1,340	55.84	51.00	29.99	**<0.001**	**0.27**
18	1,786	43.97	34.00	24.04	2,322	51.85	45.33	27.68	**<0.001**	**0.30**
19	1,605	43.17	34.00	23.88	1,795	51.36	45.33	27.59	**<0.001**	**0.32**
20	843	42.93	34.00	24.38	1,072	49.55	42.50	27.04	**<0.001**	**0.26**
21	645	43.07	34.00	24.01	768	49.74	42.50	28.14	**<0.001**	**0.25**
22	435	43.11	34.00	23.62	378	48.53	42.50	26.83	**0.004**	**0.22**
12–22	13,121	49.68	42.50	28.27	14,302	55.40	51.00	29.85	**<0.001**	**0.20**
Memory										
12	298	68.09	68.75	8.21	317	68.53	70.00	7.40	0.354	0.06
13	546	68.95	70.00	8.66	532	68.78	70.00	7.90	0.183	−0.02
14	1,767	70.22	71.50	7.84	1,946	70.57	71.50	7.34	0.362	0.05
15	2,325	71.23	71.50	7.31	2,174	71.27	71.50	6.81	0.555	0.01
16	1,567	71.47	71.50	7.83	1,619	72.09	71.50	6.65	0.109	0.09
17	1,259	72.08	71.50	7.22	1,338	72.16	71.50	6.75	0.897	0.01
18	1,793	72.56	73.00	7.13	2,321	72.61	73.00	6.76	0.827	0.01
19	1,605	73.17	73.00	6.81	1,777	73.21	73.00	6.27	0.829	0.01
20	846	72.89	73.00	7.12	1,064	73.65	73.00	6.70	0.091	0.11
21	647	72.95	73.00	7.24	765	73.64	73.00	6.94	0.219	0.10
22	436	73.05	73.00	7.71	379	73.05	73.00	6.84	0.890	0.00
12–22	13,089	71.71	71.50	7.54	14,232	72.01	71.50	6.96	0.078	0.04

*g*, Hedges’ effect size; Effect sizes of 0.20 or greater are bolded; M, mean; SD, standard deviation; *p*, alpha from Mann–Whitney U tests.

Regarding possible race-associated differences, for the Reaction Time module, the effect sizes ranged from extremely small (negligible) to small across all ages for both sexes (Table 5). The distributions of Reaction Time scores, by race, for both sexes, are almost entirely overlapping for almost every age group. There was a small magnitude difference among 22-year-old women, such that Black women had faster Reaction Time scores than White women (*g* = −0.28). Figure 2 presents overlapping density plots for cognitive module scores by race and by sex (boys/men in Panels B–E and girls/women in Panels G–J).

**Table 5:** Sway cognitive module scores stratified by age, sex, and self-identified race.

	Boys/men								Girls/women							
	White			Black					White			Black				
	*n*	M	SD	*n*	M	SD	*p*	*g*	*n*	M	SD	*n*	M	SD	*p*	*g*
Reaction time																
12	234	246.99	47.44	64	243.05	40.67	0.747	−0.09	275	260.20	47.84	45	262.64	65.79	0.548	0.05
13	467	244.60	61.30	86	245.50	54.64	0.715	0.01	469	249.86	43.72	70	241.55	43.56	0.132	−0.19
14	1,436	236.47	42.36	356	237.55	44.26	0.699	0.03	1,721	244.47	44.13	255	246.11	49.91	0.897	0.04
15	1,874	231.83	39.03	482	234.27	41.10	0.400	0.06	1,910	240.75	40.95	291	239.78	42.26	0.376	−0.02
16	1,199	231.55	39.10	376	233.41	41.66	0.638	0.05	1,399	241.42	43.20	234	243.13	52.02	0.637	0.04
17	996	229.46	39.79	278	233.06	42.16	0.221	0.09	1,158	241.38	38.58	190	242.81	43.81	0.973	0.04
18	1,371	225.21	32.25	436	226.74	34.98	0.656	0.05	2,082	237.67	34.93	252	233.99	35.61	0.052	−0.11
19	1,266	226.00	33.55	346	228.77	36.72	0.311	0.08	1,631	236.95	36.27	170	240.25	42.98	0.433	0.09
20	664	226.82	33.18	186	232.62	40.62	0.131	0.17	966	239.61	37.27	111	249.00	45.50	0.056	**0.25**
21	477	231.17	36.10	175	234.69	42.51	0.704	0.09	692	238.42	36.21	75	239.30	42.30	0.944	0.02
22	306	232.69	40.17	131	231.61	36.60	0.944	−0.03	326	244.38	37.29	54	234.01	39.66	**0.030**	**−0.28**
12–22	10,290	231.21	39.54	2,916	232.99	40.80	0.102	0.04	12,629	241.03	39.80	1,747	241.74	45.35	0.243	0.02
Impulse control																
12	234	391.20	76.00	65	387.98	67.95	0.929	−0.04	275	416.47	84.74	45	417.52	81.65	0.882	0.01
13	465	374.44	73.79	86	394.49	95.43	0.123	**0.26**	469	393.55	77.64	69	396.03	84.31	0.914	0.03
14	1,431	362.18	67.57	355	370.03	68.14	0.049	0.12	1,719	386.05	72.36	252	393.97	77.46	0.281	0.11
15	1,870	352.46	61.03	471	361.40	62.97	**0.005**	0.15	1,901	371.82	66.72	287	385.08	76.88	**0.022**	0.19
16	1,198	349.13	63.74	376	363.87	70.87	**<0.001**	**0.22**	1,397	371.19	68.06	230	377.27	79.40	0.605	0.09
17	994	340.50	62.14	274	362.85	70.78	**<0.001**	**0.35**	1,162	367.05	65.04	185	382.36	69.16	**0.003**	**0.23**
18	1,366	336.66	54.85	426	351.89	60.97	**<0.001**	**0.27**	2,083	364.26	64.84	251	372.55	75.66	0.150	0.13
19	1,261	338.34	58.04	344	351.83	63.08	**<0.001**	**0.23**	1,623	360.12	61.20	168	366.99	67.96	0.212	0.11
20	659	337.20	55.77	181	349.07	56.78	**0.005**	**0.21**	962	355.37	61.85	109	374.89	71.22	**0.008**	**0.31**
21	480	339.01	58.54	173	359.16	68.30	**<0.001**	**0.33**	692	353.87	62.24	76	368.23	68.99	0.068	**0.23**
22	304	344.80	59.96	127	349.43	60.69	0.435	0.08	327	358.18	66.61	53	351.39	55.01	0.829	−0.10
12–22	10,262	348.47	63.01	2,878	360.52	66.97	**<0.001**	0.19	12,610	369.68	67.95	1,725	380.32	75.40	**<0.001**	0.15
Inspection time																
12	234	66.51	33.02	66	69.50	32.75	0.477	0.09	273	71.97	32.68	45	72.79	36.38	0.904	0.02
13	466	62.46	33.41	87	60.57	30.79	0.757	−0.06	466	64.70	32.57	69	67.79	36.13	0.610	0.09
14	1,432	55.58	31.01	356	61.55	33.44	**0.002**	0.19	1,716	61.05	31.62	251	62.99	34.47	0.666	0.06
15	1,854	51.81	27.57	482	58.02	32.83	**0.002**	**0.22**	1,897	56.97	30.01	285	60.06	32.70	0.241	0.10
16	1,192	49.38	27.84	374	57.45	32.04	**<0.001**	**0.28**	1,396	56.70	29.45	229	54.80	30.46	0.229	−0.06
17	987	46.12	24.96	277	55.34	33.23	**0.001**	**0.34**	1,157	55.04	29.39	183	60.88	33.19	**0.046**	0.19
18	1,361	42.51	22.78	425	48.66	27.17	**<0.001**	**0.26**	2,075	51.71	27.49	247	52.97	29.25	0.727	0.05
19	1,264	42.86	23.51	341	44.34	25.19	0.532	0.06	1,628	51.19	27.24	167	53.02	30.86	0.849	0.07
20	662	43.11	24.21	181	42.30	25.03	0.445	−0.03	965	49.10	26.35	107	53.60	32.49	0.498	0.17
21	476	42.80	23.22	169	43.82	26.16	0.863	0.04	694	50.31	28.31	74	44.38	26.04	0.073	**−0.21**
22	306	42.26	22.74	129	45.11	25.57	0.377	0.12	326	49.17	27.25	52	44.46	23.92	0.274	−0.18
12–22	10,234	48.72	27.43	2,887	53.08	30.82	**<0.001**	0.15	12,593	55.15	29.50	1,709	57.25	32.30	0.148	0.07
Memory																
12	235	68.79	8.44	63	65.48	6.68	**<0.001**	**−0.41**	276	68.82	7.29	41	66.55	7.89	**0.037**	**−0.31**
13	463	69.19	8.59	83	67.63	8.99	0.110	−0.18	466	68.93	7.83	66	67.69	8.34	0.167	−0.16
14	1,420	70.51	7.84	347	69.05	7.75	**<0.001**	−0.19	1,701	70.93	7.11	245	68.08	8.37	**<0.001**	**−0.39**
15	1,858	71.72	7.22	467	69.29	7.35	**<0.001**	**−0.34**	1,891	71.48	6.76	283	69.90	6.98	**<0.001**	**−0.23**
16	1,198	72.24	7.78	369	68.97	7.46	**<0.001**	**−0.42**	1,390	72.34	6.61	229	70.60	6.73	**<0.001**	**−0.26**
17	993	72.48	7.23	266	70.61	6.98	**0.005**	**−0.26**	1,153	72.38	6.78	185	70.82	6.43	**0.005**	**−0.23**
18	1,365	73.04	7.21	428	71.04	6.64	**<0.001**	**−0.28**	2,073	72.80	6.76	248	71.05	6.56	**<0.001**	**−0.26**
19	1,263	73.72	7.00	342	71.16	5.63	**<0.001**	**−0.38**	1,615	73.29	6.26	162	72.48	6.35	0.069	−0.13
20	662	73.50	7.10	184	70.68	6.75	**<0.001**	**−0.40**	957	73.83	6.62	107	72.00	7.23	**0.004**	**−0.27**
21	478	73.52	7.25	169	71.35	6.98	**<0.001**	**−0.30**	693	73.76	6.87	72	72.44	7.49	**0.024**	−0.19
22	307	74.37	7.36	129	69.92	7.66	**<0.001**	**−0.60**	326	73.33	6.75	53	71.33	7.20	**0.004**	**−0.29**
12–22	10,242	72.21	7.56	2,847	69.34	7.18	**<0.001**	**−0.38**	12,541	72.23	6.89	1,691	70.37	7.26	**<0.001**	**−0.27**

*g*, Hedges’ effect size; Effect sizes of 0.20 or greater are bolded; M, mean; SD, standard deviation; *p*, alpha from Mann–Whitney U tests.

For the Impulse Control module, the effect sizes for race-associated differences ranged from extremely small (negligible) to small across all ages and within both sexes (Table 4). There were small differences between groups, such that White boys/men had faster scores than Black boys/men at the following ages: age 14 (*g* = 0.12), age 15 (*g* = 0.15), and ages 16–21 (*g*’s = 0.21–0.35). There was also a small difference for girls/women such that White girls/women had faster scores than Black girls/women at the following ages: age 15 (*g* = 0.19), age 17 (*g* = 0.23), and age 20 (*g* = 0.31).

For the Inspection Time module, the effect sizes for race-associated differences ranged from extremely small (negligible) to small across all ages and within both sexes (Table 4). The distributions of Inspection Time scores, by race, for both sexes, are almost entirely overlapping for most age groups. There was a small effect size difference for 14 (*g* = 0.19), 15 (*g* = 0.22), 16 (*g* = 0.28), 17 (*g* = 0.34) and 18-year-old boys (*g* = 0.26), with White boys having faster scores than Black boys. White girls had faster scores than Black girls for the 17-year-old age group (*g* = 0.19).

For the Memory module, the effect sizes for race-associated differences ranged from small to medium across all ages and within both sexes (Table 4). White boys had higher Memory scores than Black boys (*g* = −0.19 to −0.42) for all ages, with the exception of age 13 (*p* > 0.05; *g* = −0.18), and White men had higher Memory scores than Black men (*g* = −0.30 to −0.60). White girls had higher Memory scores than Black girls (*g* = −0.16 to −0.39) for all ages except age 13 (*p* > 0.05; *g* = −0.16) and White women had higher Memory scores than Black women (*g* = −0.13 to −0.29) for all ages except age 19 (*p* > 0.05; *g* = −0.13).

## 4 Discussion

This study investigated possible race-associated differences in Sway Medical System Balance and Cognitive module scores among student athletes undergoing preseason baseline testing. There is a lack of literature examining race-associated differences in Balance assessments in adolescent and college-aged athletes and this has been identified as a need for future research (28). The current study adds new information to the field about the negligible race-associated differences in Balance scores on the Sway Medical System Balance module. The negligible race-associated differences observed in our study, particularly in contrast to larger differences reported on other neuropsychological assessments, may reflect factors relating to the nature of the Sway assessment modules, discussed more below.

In general, the findings did not support our hypothesis that student-athletes who self-identified as Black or African American would have lower cognitive test scores, on average, than those who identified as White. Although statistically significant, the race-associated differences in cognitive scores, such as Reaction Time, Impulse Control, and Inspection Time were negligible in terms of effect size magnitude. It is possible that the minimal differences observed on these timed cognitive modules reflect the nature of these tasks, which primarily assess basic processing speed and motor response time rather than complex cognitive abilities, which may be more susceptible to educational, cultural, or sociodemographic influences. One prior study by Farah and colleagues showed that children in higher SES groups performed better across neurocognitive tests, but that this difference was non-uniform, where the difference was larger in the more complex task (i.e., tasks that involved the language system), compared to more simple tasks (i.e., reward processing and visual cognition), implying that this difference depended on the task and complexity of the task (35). The race-associated differences in Memory scores had small to medium effect sizes. As can be seen by visual inspection of Figures 2E,J, despite small to medium effect sizes the overall distribution of Memory scores is more similar than it is different between those who self-identify as White vs. those who self-identify as Black. This suggests that these results may not be practically or clinically meaningful. The small to medium effect sizes for Memory scores, while larger than other cognitive modules, may be due in part to greater complexity of memory tasks compared to simple reaction time measures, though the extensive overlap in score distributions suggest limited clinical significance.

Additionally, we hypothesized that there would be no race-associated differences in the Sway System Balance module scores, which was supported by findings in the current study. It is possible that SES and other social determinants of health are minimally associated with scores on the Balance module in student athletes. The Balance scores between Black and White girls/women and Black and White boys/men were not significantly different for most ages, with the exception of a small effect size difference for 13-year-old boys and a small effect difference for 19-year-old women. Specifically, 13-year-old Black boys had slightly higher balance scores than 13-year-old White boys, with a small effect size (*g* = 0.29) and 19-year-old White girls had slightly higher balance scores than 19-year-old Black girls. We do not have an explanation for these small differences; they could be related to sampling or they could be spurious. Overall, the distributions of Balance module scores for Black and White individuals were almost entirely overlapping for every age group, except for a small difference for 13-year-old boys and 19-year-old girls. This suggests that these results may not be practically or clinically meaningful.

Several prior studies have examined race-associated differences in cognitive tests that are used as part of the medical management of concussions (18, 28, 36–38). Prior literature has noted that White athletes had faster Reaction Time scores, on ImPACT baseline testing, compared to their Black peers (18, 28). In addition, White high school (38) and college (28) athletes scored significantly lower (i.e., faster speed) than Black athletes on the Visual Motor Speed composite of ImPACT at baseline. Further, race-associated differences in Visual and Verbal Memory composite scores in high school athletes at baseline have been reported (18). In contrast, one study found no race-associated differences on cognitive scores on ImPACT at baseline (37).

In the current study, the sex-associated differences in Reaction Time (*g* = 0.17 to 0.34), Impulse Control (*g* = 0.17 to 0.40), and Inspection Time (*g* = 0.09 to 0.32) were small, with boys/men having slighter faster scores than girls/women. On Sway, the Reaction Time, Impulse Control, and Inspection Time modules are measured in milliseconds and are all interpreted the same way, where lower scores are faster times and are better, compared to higher, slower scores which are worse (39). Past studies also have shown a sex difference in timed assessments (e.g., simple reaction time) in samples ranging from children to young adults (40–45). The three timed cognitive modules of the Sway Medical System appear to be consistent with the previous literature on simple reaction time.

### 4.1 Limitations and future research

There are several limitations to the current study. First, participants included 12- to 22-year-old student-athletes, so findings may not be generalizable to other populations, including older adults and people who are not athletes. Second, the data used for this study was part of a large population dataset, and the researchers did not oversee data collection. It is possible that some participants were tested remotely using their personal device (i.e., smartphone or tablet), and it is unknown if scores differ by environment and administrator presence. Third, there are limitations in comparing findings to other studies using different cognitive and/or balance testing batteries. Notably, the Sway Medical System’s Balance Module protocol involves variations from the widely used Balance Error Scoring System (BESS) assessment. Specifically, the Sway protocol requires single-leg testing performed on each leg and two tandem stance trials, with alternating front feet, compared to the BESS, which requires participants to complete one trial of each of the three stances (i.e., single leg on the non-dominant foot, double leg with feet together, and tandem stance with the dominant foot in front). Additionally, they differ by testing surface (foam board used in BESS), and timing on assessments (Sway requires each stance for 10 s, versus 20 s per stance on BESS). Therefore, our findings regarding Balance are only applicable to Sway Medical System’s Balance testing and not to the BESS or modified BESS.

Fourth, data were not available regarding other neurodevelopmental conditions or a history of individualized education programs (IEPs), which could potentially influence cognitive test performance and should be considered in future studies examining possible race-associated differences in neuropsychological assessments. Fifth, additional demographic and sports-related variables that could influence test performance were not available for analysis, including total number of previous concussions, type and number of sports played, school academic rankings, parental education levels, detailed socioeconomic status measures, and area deprivation index scores. Future research could incorporate these variables to better understand potential mediating factors in race-associated differences in cognitive and balance assessments. Finally, our study examined race-associated differences for those who identify as White or those who identify as Black or African American. Future studies should determine if there are race-associated differences in other races—and if so, attempt to deconstruct the factors associated with those differences (e.g., SES, stereotype threat, cultural bias) (46).

## 5 Conclusion

The current study examined potential race-associated differences in Sway Medical System Balance and Cognitive module scores during preseason baseline testing. There were no clinically meaningful race-associated differences between student-athletes who self-identified as Black and those who identified as White for Balance, Reaction Time, Impulse Control, and Inspection Time. It is possible that scores derived from the Sway cognitive modules, especially Reaction Time, Impulse Control, and Inspection Time, might be less influenced by education, quality of education, parental education, culture, SES, and other psychosocial factors compared to traditional face-to-face office-based neuropsychological testing, (assuming, of course, similar access and exposure to mobile phone use). They might be less influenced by SES and other social determinants of health because, at least in part, they measure simple reaction time, choice reaction time, and basic processing speed versus cognitive domains that are more multifaceted and influenced by quality of education and cultural experiences. Memory score differences were small to medium. The reasons for small race-associated differences in Memory scores are unknown and future research to examine the possible role or influence of social risk factors and psychosocial factors on test scores is recommended.

## Data Availability

The data presented in this article were provided to the authors through a research agreement with Sway Medical, LLC. The original contributions presented in the study are included in the article. The data used for this study are not publicly available and are not included in a repository. Requests to access the datasets should be directed to GI, giverson@mgh.harvard.edu.
